# Time-homogeneous Markov process for HIV/AIDS progression under a combination treatment therapy: cohort study, South Africa

**DOI:** 10.1186/s12976-017-0075-4

**Published:** 2018-01-18

**Authors:** Claris Shoko, Delson Chikobvu

**Affiliations:** Department of Mathematical Statistics and Actuarial Sciences, University of the Free State, Box 339, Bloemfontein, 9300 South Africa

**Keywords:** HIV/AIDS progression, Homogeneous Markov models, Reaction to treatment

## Abstract

**Background:**

As HIV enters the human body, its main target is the CD4 cell which it turns into a factory that produces millions of other HIV particles. These HIV particles target new CD4 cells resulting in the progression of HIV infection to AIDS. A continuous depletion of CD4 cells results in opportunistic infections, for example tuberculosis (TB). The purpose of this study is to model and describe the progression of HIV/AIDS disease in an individual on antiretroviral therapy (ART) follow up using a continuous time homogeneous Markov process. A cohort of 319 HIV infected patients on ART follow up at a Wellness Clinic in Bela Bela, South Africa is used in this study. Though Markov models based on CD4 cell counts is a common approach in HIV/AIDS modelling, this paper is unique clinically in that tuberculosis (TB) co-infection is included as a covariate.

**Methods:**

The method partitions the HIV infection period into five CD4-cell count intervals followed by the end points; death, and withdrawal from study. The effectiveness of treatment is analysed by comparing the forward transitions with the backward transitions. The effects of reaction to treatment, TB co-infection, gender and age on the transition rates are also examined. The developed models give very good fit to the data.

**Results:**

The results show that the strongest predictor of transition from a state of CD4 cell count greater than 750 to a state of CD4 between 500 and 750 is a negative reaction to drug therapy. Development of TB during the course of treatment is the greatest predictor of transitions to states of lower CD4 cell count. Transitions from good states to bad states are higher on male patients than their female counterparts. Patients in the cohort spend a greater proportion of their total follow-up time in higher CD4 states.

**Conclusion:**

From some of these findings we conclude that there is need to monitor adverse reaction to drugs more frequently, screen HIV/AIDS patients for any signs and symptoms of TB and check for factors that may explain gender differences further.

## Background

The life cycle of HIV starts as it enters the human body, its major target being a white blood cell called T-helper cells or CD4 cells [1]. Once these cells are infected, HIV takes over and turns them into factories that produce thousands of copies of the virus. The HIV makes use of the enzyme Reverse Transcriptase to change copies of its Ribonucleic Acid (RNA) into Deoxyribose nucleic Acid (DNA). The viral DNA then enters the nucleus of the host cell and combines with cell DNA and starts making copies of viral RNA. The enzyme protease helps in assembling the viral particles into thousands of new viruses, which will bud and destroy the host cell. These new viruses will then be ready to attack other CD4 + T cells. Hence, the importance of CD4+ T cell count in understanding the progression of HIV. Depreciation of the CD4+ T cells in the human body leads to the deterioration of the human immune system, which is why the virus is called the Human Immunodeficiency Virus (HIV).

As the immune system is compromised, the individual is now prone to opportunistic infections like tuberculosis (TB). TB may occur at any stage of HIV disease and is frequently the first recognised presentation of underlying HIV [2]. HIV and TB coinfection is characterised by challenges that include poor adherence and overlapping toxicities resulting in an impaired CD4 T-cell recovery with antiretroviral therapy (ART) due to the effect of drug-drug interaction [3].

The need to address the challenges associated with HIV/AIDS progression in the presence of TB coinfection has prompted this study and also to analyse HIV disease history based CD4 multi-states and death/loss to follow-up in a single model. However, most studies use Kaplan-Meier analysis and Cox proportional hazards regression models [3–5]. Kaplan-Meier survival methods and Cox proportional hazards regression are commonly employed tools to model mortality and time to viral suppression and/or subsequent rebound and occasionally used to model time to CD4 recovery. However, survival models are not appropriate for all studies, particularly in the presence of competing risks and when multiple or recurrent outcomes are of interest. In particular, when modelling HIV/AIDS progression, Markov models are relatively straight forward to analyse both CD4 stage and death or loss to follow-up within a single model which survival models fail to do. Markov models can accommodate censored data, competing risks (informative censoring), multiple outcomes, recurrent outcomes, frailty and non-constant survival probabilities [6]. Examination of the conditions of the stochastic processes at various points in time, categorisation of the conditions, and examination of the external influences on the stochastic processes can be done using Markov models [7]. Markov models are favourable to the modelling of diseases in particular cases where the disease is grouped into a set of exhaustive and mutually exclusive health states, thereby forming a multi-state model [8]. History is naturally generated as the multi-states evolve over time; it contains information on previous visits, time of entry into various states, and the length of stay in states.

Continuous-time homogeneous Markov models have been used since early in the epidemic to model disease progression of HIV/AIDS patients, and there has been some recent renewed interest in the use of these models. In 1989 Longini et al. used a 5-state Markov model based on the clinical indicators of the HIV disease progression [9]. In 1998 Alioum et al. estimated the effects of gender, age, mode of transmission and ART on HIV progression using a 3-state Markov model [10]. In 2011 Reddy [11] carried out a research almost similar to Alioum et al. in South Africa. However, Reddy used a 5-state Markov model with 4 CD4 based transient states followed by the absorbing state, ARV initiation. Reddy’s model is characterised by high rates of immune deterioration since the study was carried out on ARV naïve patients. In 2009, Binquet et al. used a multi-state Markov model to analyse the impact of gender, intravenous drug use, weight loss, low haemoglobin, CD8 cell count and HIV viral load on HIV evolution in the era of highly active antiretroviral therapy (HAART) [12]. Recently, in 2013 Grover et al. assessed the impact of ART using a 5-staged multistate Markov model and went further to examine the effects of explanatory variables; age, sex and mode of transmission on the transition rates [13].

In this study, we use 7-staged continuous-time Markov model to assess the disease progression of HIV/AIDS patients receiving ART from a clinic in Bela-Bela, South Africa. The first 5 stages are based on CD4 cell counts and the end points are either death or withdrawal from study. In addition to the gender and age differences in HIV/AIDS progression, we further assess the effects of having TB as the initial marker of HIV/AIDS, developing TB during the course of treatment, developing some adverse effects to treatment (Reaction), CD4 baseline and viral load baseline. Though Markov models based on CD4 cell counts is a common approach in HIV/AIDS modelling, this paper is unique clinically in that tuberculosis (TB) co-infection is included as a covariate.

In medical research, the state of the patient at observation time is the only thing known with certainty. The researcher may know the time interval in which a transition has occurred, but not the exact time. Thus, homogeneous Markov models which are interval censored can handle such data [14]. The transition intensities, probabilities and the distribution functions associated with the times are the basic building blocks of the Markov processes [15]. For a continuous-time Markov model, transitions can occur at any (real-valued) time instant. For a time-homogeneous Markov jump process, the holding time in state i are modelled using exponential distributions. The exponential distributions may be adequate for many real-life situations, for example, time until death, and waiting time before moving to another state. However, the exponential distributions are memoryless continuous distributions, hence a limitation in the application of Markov processes. The ‘memoryless’ property could be seen as a problematic assumption in this setting. It is likely the case that patients starting on ART who respond well to treatment will continue to respond well to treatment - contradicting the Markov assumption and memoryless property.

Transition probabilities for continuous-time homogeneous models only depend on the difference between the two observation times. That is, for all *t* ≥ 0 the probability of moving from state *i* to state *j* is given by:

$$ {p}_{ij}\left(s,t\right)=P\left[{X}_t=j|{F}_t\right]=P\left({X}_t=j|{X}_s=i\right)=P\left({X}_{t-s}=j|{X}_0=i\right),\forall t\ge 0,t>s. $$

This is the Markov property, where *F*_*t*_ is the natural Filtration of the stochastic process. *P*[*X*_*t*_ = *j*| *F*_*t*_], therefore, represents the probability that the stochastic process *X*_*t*_ is in state *j* at time *t* given the history of the process up to time *t*. The Markov property implies that all the history of the process is contained in the state currently occupied, *X*_*s*_ = *i*. The transition probabilities of a continuous time homogeneous Markov process *X*_*t*_, *t* ≥ 0 is given by:

$$ {p}_{ij}(t)=P\left({X}_t=j|{X}_0=i\right) $$

The equations obey the Chapman-Kolmogorov equations:

1 $$ {p}_{ij}\left(t+s\right)={\sum}_{k\in X}{p}_{ik}(s){p}_{kj}(t)\kern1.25em \forall s,t>0 $$

In this paper we describe, using the theory of continuous time Markov processes, and using real data on an evolving disease such as AIDS. Also, the effects of covariates, including TB, on baseline transition rates is considered. Models with and without covariates are fitted and compared using the likelihood ratio test.

The next section explores the methods of Markov modelling and an illustrative case study on HIV progression is given. In this section, data used in the analysis is described and we explain formulation of the model based on the data. This is followed by a section on the results and discussions. The final section concludes on the findings.

## Methods

### A continuous-time homogeneous Markov model

Formulation of the continuous-time homogeneous model is done by considering transition probabilities over narrow interval of time *∆t*. In this study $\Delta  t=\frac{1}{2}$ year making it appropriate to assume that transition rates over these intervals are constant. These transition rates, also known as transition intensities or forces of transition, are the fundamental concept in continuous time Markov jump processes. They can take values greater than 1, unlike probabilities. In order to differentiate the transition probabilities and avoid technical problems with mathematics, the assumption is that the functions *p*_*ij*_(*t*) are continuously differentiable and are subject to the initial condition:

2 $$ {p}_{ij}(0)={\delta}_{ij}=\left\{\begin{array}{c}0\  if\ i\ne j\\ {}1\  if\ i=j\end{array}\right. $$

*δ*_*ij*_ is a Kronecker delta, *p*_*ii*_(0) = 1 means that at *t* = 0 the system maintains its original state and *p*_*ij*_(0) = 0 means that there is no change of state when no time elapses. The force of transition from state *i* to *j* is defined as:

$$ {\left.{\alpha}_{ij}=\frac{d}{dt}{p}_{ij}(t)\right|}_{t=0}=\underset{\Delta t\to 0}{\lim}\frac{p_{ij}\left(\Delta t\right)-{\delta}_{ij}}{\Delta t} $$

*α*_*ij*_, for *i* = 1, …, 5 and *j* = 1, …, 7, does not vary over time and satisfies the following conditions; ${\sum}_{j\in X}{\alpha}_{ij}=0$ and ${\alpha}_{ii}=-{\sum}_{i\ne j}{\alpha}_{ij}$.

Once the transition intensities are known, the transition probabilities can be obtained by solving a system of differential equations known as the Kolmogorov’s forward equation subject to the initial conditions stated in eq. (2). The Kolmogorov’s forward equation is as follows:

3 $$ \frac{d}{dt}{p}_{ij}(t)={\sum \limits}_{\forall k}{p}_{ik}(t){\alpha}_{kj}\kern1.25em \forall i,j $$

where *k* is a state that the system can pass through as it makes a transition from state *i* to state *j*. The time homogeneous models are fitted for this data to assess effectiveness of the treatment by comparing the forward transition and the reverse transitions. This then lead to building of models that allow transitions in both directions.

### Data description

The model is initially applied on 319 HIV patients on anti-retroviral therapy (ART) from a Wellness clinic in Bela Bela, South Africa, from year 2005 to year 2009. Two hundred and twenty-seven of these patients were females and 92 were males at treatment commencement (*t* = 0). After 3 years of treatment uptake, 173 females and 71 males were remaining in the study. Thirty-eight females had died and 16 withdrew and their status was not known after 3 years of treatment up take. Nineteen of the males died during the first three years and two had withdrawn and it was not known whether they were alive or dead. A 2-year old (subject 81) together with subject 82 were detected by the residuals plot as an outlier and it was removed from the analysis meaning that the remaining 317 patients were used for analysis. About 50 and 65% of the female and male deaths respectively occurred during the first 6 months of treatment uptake. The interquartile range of patient ages is (33; 47.5) years with a mean and median age of 39.53 and 40 years respectively. The ages were negatively skewed (skew = −0.24) which means that there were more younger patients than older patients in this cohort.

At time *t* = 0 there were 242 individuals with CD4 baseline (CD4BL) cell count below 200, 59 individuals with CD4 cell count between 200 and 350, 11 individuals with CD4 cell count between 350 and 500, 6 individuals with CD4 cell count between 500 and 750 and 1 individual with CD4 cell count above 750. At t = 0 the CD4 cell count had mean of 156 cells/mm^3^, a median of 116 cell/mm^3^ and the maximum CD4 cell count was 1202 cells/mm^3^. The mean viral load baseline (VLBL) for these patients was 105,573.35 copies/mm^3^ and it ranged from 56 to 818,600 copies/mm^3^. The median viral load was 58,523.00 copies/mm^3^. From these individuals 155 had a WHO stage baseline (WSBL) of 4 which is related to severe HIV symptoms. WSBL is the categorisation of HIV/AIDS at baseline basing on the clinical markers as defined by World Health Organisation (WHO).

Although some individuals developed TB (DTB) during the course of treatment, 109 patients had TB as an initial marker of HIV. From the individuals who had TB before (TBB4) commencement of antiretroviral therapy (ART), 66 had a CD4 baseline below 200cells/mm^3^, 20 had a CD4 baseline between 200 and 350cells/mm^3^, 2 had CD4 baseline between 350 and 500cells/mm^3^, 2 between 500 and 750cells/mm^3^ and 19 had unknown CD4 baseline. These patients completed their TB treatment before commencement of ART. Fifty-two patients developed TB during the treatment period and 12 of these patients had TB before commencement of treatment. During the first 6 months of treatment uptake, 35 patients died and from these deaths, only five were attributed to having TB before commencement of ART.

A combination therapy was administered to all HIV-infected individual in the cohort. The therapeutic intervention inhibits the actions of reverse transcriptase enzyme and/or protease of new infectious free HIV by the HIV-infected cell. The drug regimens at *t* = 0 were mainly a combination of d4T-3TC-EFV (administered to 207 patients) and d4T-3TC-NVP (administered to 83 patients). The second line regimens were mainly a combination of AZT-3TC-EFV/NVP and were given to patients who developed some adverse reaction. These second line regimens were frequently used from 2 to 4 years post-treatment commencement. The therapeutic intervention lowers the number of infectious free virus particles in the circulation, and in some cases to beyond detection. This results in a reduction on the density of infected cells, causing a rise on the CD4 cell count of infected individuals. So generally the CD4 cell count of an individual receiving therapeutic intervention is expected to rise to well above 500 cell/mm^3^, assuming a proper adherence to treatment. Hence the use of increase in CD4 cell count as the marker of efficacy of treatment.

During the course of treatment, some individuals developed some adverse reaction (React) to treatment. For these individuals the adverse reactions were treated and drugs administered to them were changed. Change of treatment was also based on the viral load monitoring.

For the purpose of analysis, the variables are coded into the model as follows:

**Table:** 

	WSBL	Gender	Age	CD4BL	VLBL	DTB	TBB4	React
1	4	Male	≤ 40*years*	≤ 350	≥10 000	Yes	Yes	Yes
0	other	Female	> 40*years*	> 350	< 10 000	No	No	No

### Model formulation

At any time *t* + *∆t*, the state of an HIV-infected individual is defined basing on the CD4 cell count level or whether the individual is dead or has withdrawn as follows:

**Table:** 

**State 1**-*CD*4 ≥ 750	**State 2**–500 ≤ *CD*4 < 750
**State 3**–350 ≤ *CD*4 < 500	**State 4**–200 ≤ *CD*4 < 350
**State 5**-*CD*4 < 200	**State 6**-Death
**State 7**-Withdrawal.	

**Fig. 1:**
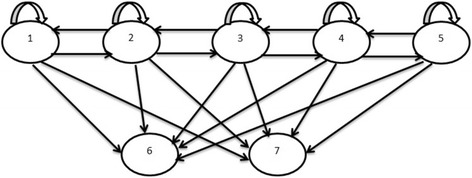
The State Diagram for HIV Progression of Individuals on ART

Basing on these seven states, progression of HIV positive individuals on treatment is defined by the state diagram on Fig. 1 below. The arrows in the diagram show possible transitions between the seven states defined above.

The information in Fig. 1 shows that state 6 and 7 are absorbing states hence no transitions from these states. As HIV progresses in an individual’s body, there is a possibility of an individual being in the same state in consecutive visit times.

Basing on the classification above, Table 1 summarises transition counts that took place for the whole period of study 2005 to 2009.

**Table 1:** Transition Counts from 2005 to 2009

	To	1	2	3	4	5	6	7
From	1	69	34	5	1	1	1	1
	2	47	80	37	4	0	2	4
	3	21	79	193	46	9	6	5
	4	0	14	128	203	37	8	9
	5	0	3	26	117	204	42	8

Table 1 shows that, transition counts from state *i* to *i* ± *j* are higher for all the values in which *j* = 1 than for *j* > 1 where *i*, *j* ∈ { 1, …, 5}. As a result a bidirectional model is proposed which defines transitions from state *i* to *i* ± 1 or from *i* to *j* = 6; 7.

The model is formulated basing on the assumptions that between times (*t*, *t* + *∆ t*), where *∆t* is a very small value, there is a transition from any one of the states *i* = 1, 2, …, 5 (transient states) to state *j* = 1, 2, …, 7 defined as follows:

- CD4 cell count of an individual is expected to rise due to efficacy of treatment at a rate of *α*_*ij*_,  where *j* = *i* − 1;
- Some individuals fail to adhere to treatment therapy. These individuals can move to a state of lower CD4 cell count at a rate of *α*_*ij*_,  where *j* = *i* + 1;
- From any state *i* = 1, 2, …, 5 an infected individual can die (state 6) at a rate of *α*_*i*6_;
- An individual in state *i* = 1, 2, …, 5 can decide to withdraw (state 7) at a rate of *α*_*i*7_;
- An individual can remain in the same state at a rate of *α*_*ii*_ =  − *λ*_*i*_ =  − (*α*_*i*, *i* − 1_ + *α*_*i*, *i* + 1_ + *α*_*i*6_ + *α*_*i*7_). This is based on the fact that the sum of transition rates from any state is equal to zero.

These assumptions can be represented by the following transition rate matrix *Q*(*t*):

$$ Q(t)=\left(\begin{array}{ccccccc}-\left({\alpha}_{12}+{\alpha}_{16}+{\alpha}_{17}\right)& {\alpha}_{12}& 0& 0& 0& {\alpha}_{16}& {\alpha}_{17}\\ {}{\alpha}_{21}& -\left({\alpha}_{21}+{\alpha}_{23}+{\alpha}_{26}+{\alpha}_{27}\right)& {\alpha}_{23}& 0& 0& {\alpha}_{26}& {\alpha}_{27}\\ {}0& {\alpha}_{32}& -\left({\alpha}_{32}+{\alpha}_{34}+{\alpha}_{36}+{\alpha}_{37}\right)& {\alpha}_{34}& 0& {\alpha}_{36}& {\alpha}_{37}\\ {}0& 0& {\alpha}_{43}& -\left({\alpha}_{43}+{\alpha}_{45}+{\alpha}_{56}+{\alpha}_{57}\right)& {\alpha}_{45}& {\alpha}_{56}& {\alpha}_{57}\\ {}0& 0& 0& {\alpha}_{54}& -\left({\alpha}_{54}+{\alpha}_{56}+{\alpha}_{57}\right)& {\alpha}_{56}& {\alpha}_{57}\\ {}0& 0& 0& 0& 0& 0& 0\\ {}0& 0& 0& 0& 0& 0& 0\end{array}\right) $$

Once the transition rate matrix has been obtained, the matrix of transition probabilities can be obtained using Kolmogorov’s forward differential equations defined in (3). This yields the following differential equations for the Markov jump processes:

4 $$ \frac{dp_{i1}(t)}{dt}=-\left({\alpha}_{12}+{\alpha}_{16}+{\alpha}_{17}\right){p}_{i1}(t)+{\alpha}_{21}{p}_{i2}(t)\kern1em \mathrm{for}\kern0.5em i=1,2; $$

5 $$ \frac{dp_{i2}(t)}{dt}={\alpha}_{12}{p}_{i1}(t)-\left({\alpha}_{21}+{\alpha}_{23}+{\alpha}_{26}+{\alpha}_{27}\right){p}_{i2}(t)+{\alpha}_{32}{p}_{i3}(t)\kern0.5em \mathrm{for}\kern0.5em i=1,2,3; $$

6 $$ \frac{dp_{i3}(t)}{dt}={\alpha}_{23}{p}_{i2}(t)-\left({\alpha}_{32}+{\alpha}_{34}+{\alpha}_{36}+{\alpha}_{37}\right){p}_{i3}(t)+{\alpha}_{43}{p}_{i4}(t)\kern0.5em \mathrm{for}\kern0.5em i=2,3,4; $$

7 $$ \frac{dp_{i4}(t)}{dt}={\alpha}_{34}{p}_{i3}(t)-\left({\alpha}_{43}+{\alpha}_{45}+{\alpha}_{46}+{\alpha}_{47}\right){p}_{i4}(t)+{\alpha}_{54}{p}_{i5}(t)\kern0.5em for\kern0.5em i=3,4,5; $$

8 $$ \frac{dp_{i5}(t)}{dt}={\alpha}_{45}{p}_{i4}(t)-\left({\alpha}_{54}+{\alpha}_{56}+{\alpha}_{57}\right){p}_{i5}(t)\kern0.5em \mathrm{for}\kern0.5em i=4,5; $$

9 $$ \frac{dp_{i6}(t)}{dt}={\sum}_{k=1}^5{p}_{ik}(t){\alpha}_{k6}\kern0.5em \mathrm{for}\kern0.5em i=1,\dots, 5; $$

10 $$ \frac{dp_{i7}(t)}{dt}={\sum}_{k=1}^5{p}_{ik}(t){\alpha}_{k7}\kern0.5em \mathrm{for}\kern0.5em i=1,\dots, 5. $$

Equations (4) to (10) represent all the possible transition probabilities from state *i*, for *i* = 1, 2, ...5, to state *j* = 1, …, 7. *p*_*ij*_(*t*) represents the probability that a patient in state *i* makes a transition to state *j* and its coefficients represent the transition rates. For example, in equation (4), −(*α*_12_ + *α*_16_ + *α*_17_) = *α*_11_. These states denoted by *i* are defined based on the CD4 cell count grouping. So there is a possibility of a backward or forward movement transition between transient states due to failure or efficacy of treatment respectively. There is no possible transition from state *i* = 6 and state *i* = 7 because these states are absorbing states where *i* = 6 represents death of an infected individual and state *i* = 7 represents withdrawal from treatment by an infected individual. All the analysis is done using the package ‘msm’ for multistate modelling in R software. The package was developed by Jackson in 2011 [16].

## Results and discussions

### Estimation of the transition rate matrix

Estimation of the transition intensities is done using the method of maximum likelihood to estimate the transition intensities. The likelihood, L, is given by:

11 $$ L={e}^{\alpha_{11.}{t}_1+{\alpha}_{22.}{t}_2+\dots +{\alpha}_{77.}{t}_7}\times {\alpha}_{11}^{n_{11}}{\alpha}_{12}^{n_{12}}\dots {\alpha}_{77}^{n_{77}}, $$

where *t*_*i* _ : *i* = 1, 2, …, 7, is the total number of observed waiting/holding time in state *i*, ${\alpha}_{ii}=-{\sum}_{i\ne j}{\alpha}_{ij}$ and *n*_*ij*_ is the number of transitions observed from state *i* to state *j*. The estimates are obtained by taking the logarithm of the likelihood and differentiating this with respect to each of the transition intensities *α*_*ij*_'s. This leads to the maximum likelihood estimates of the transition intensities as ${\alpha}_{ij}=\frac{n_{ij}}{t_i}$, where *n*_*ij*_ is the number of transitions from state *i* to state *j*, and *t*_*i*_ the total observed waiting/holding time in state *i*.

**Fig. 2:**
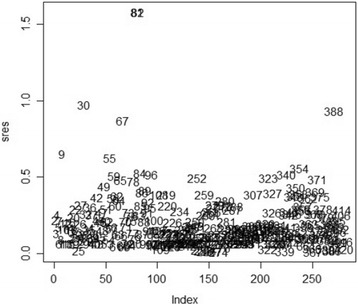
The score residuals plot for detecting outliers

The plot of residuals for each of the individuals in the study was drawn to identify the outliers (subjects with higher influence) in the data. Once the outliers are identified they can simply be deleted and the model is re-fit. According to Titman in 2007 [17] residuals for multi-state models can be determined as follows;

If *n* subjects and a parameter vector *θϵ*Θ, with maximum likelihood estimator based upon the whole data $\widehat{\theta}$. Let ${\widehat{\theta}}_{(j)}$ represent the estimate with subject *j* deleted. Thus the quantity ${\widehat{\theta}}_{(j)}-\widehat{\theta}$ for *j* = 1, …, *n* is of interest. The influence of each point on each parameter can be compared separately and to get a measure of the overall influence of a particular subject we take the scalar quantity;

$$ {\left({\widehat{\theta}}_{(j)}-\widehat{\theta}\right)}^{\hbox{'}}I\left(\widehat{\theta}\right)\left({\widehat{\theta}}_{(j)}-\widehat{\theta}\right) $$

where *I*(*θ*) is the observed Fisher information matrix at the maximum likelihood estimates for the full data. Consider the contribution to the score function of each subject evaluated to the maximum likelihood estimate for the full model. Highly influential subjects will have scores of high magnitude. For a single subject, the score residual is given by an analogous scalar measure:

$$ {U}_j{\left(\widehat{\theta}\right)}^{\hbox{'}}I{\left(\widehat{\theta}\right)}^{-1}{U}_j\left(\widehat{\theta}\right) $$

where ${U}_j\left(\widehat{\theta}\right)$ is the vector of first derivatives of the log-likelihood for that subject at maximum likelihood estimates *θ*. That is, $U\left(\theta \right)=\frac{\partial l}{\partial \theta}\left(\theta \right),$ is determined using the derivative of the transition probability matrix *P*(*t*) with respect to *θ*. These derivatives were given by Kalbfleisch and Lawless [18]. The residuals plot displays the residuals for each subject in the order labelled by subject identifiers. Subjects with a higher influence on the maximum likelihood estimates will have higher score residuals [16]. The plot helps to identify any outliers in the data. Figure 2 below shows the plot of residuals.

Results from Fig. 2 show that patients with ID numbers 81 and 82 are outliers as indicated by their positions from the rest of the patients in the cohort. The corresponding residuals for these values are 1.58315799 and 1.58315999, respectively compared to the rest of the subjects whose residuals below 1. Patient number 81 is a 2 year old enrolled whilst in state 1 and maintained the state throughout the study period. Patient number 82 was enrolled whilst in state 5 and, during the third visit, was already in state 1 and maintained it throughout the study period. These patients are excluded from the analysis leaving us with 317 subjects. Table 2 shows transition intensities *α*_*ij*_ for *i* = 1, 2, …, 5 and *j* = 1, 2, …, 6, 7 for the two models, one with outliers and the other one without outliers. The corresponding confidence interval is also given for each transition intensity. The state space is *X*_*t*_ = {1, 2, 3, 4, 5, 6, 7}.

**Table 2:** Transition intensities and their corresponding confidence intervals for the model with and the model without outliers

	With outliers	Without outliers
*α*_12_	0.9820 (0.6695,1.4410)	0.8872 (0.6355,1.2390)
*α*_16_	0.1176 (0.0678,0.2040)	0.1016 (0.0541,0.1907)
*α*_17_	0.1153 (0.0663,0.2005)	0.1418 (0.0843,0.2386)
*α*_21_	0.6871 (0.4814,0.9808)	0.5183 (0.3723,0.7217)
*α*_23_	0.4256 (0.3129,0.5790)	0.4959 (0.3729,0.6593)
*α*_26_	0.0726 (0.0406,0.1300)	0.0811 (0.0468,0.1404)
*α*_27_	0.0261 (0.0093,0.0734)	0.0377 (0.0167,0.0850)
*α*_32_	0.4373 (0.3605,0.5305)	0.4231 (0.3451,0.5186)
*α*_34_	0.3382 (0.2639,0.4335)	0.3324 (0.2605,0.4241)
*α*_36_	0.0273 (0.0127,0.0587)	0.0145 (0.0047,0.0444)
*α*_37_	0.0298 (0.0155,0.0574)	0.0311 (0.0166,0.0584)
*α*_43_	0.5524 (0.4693,0.6503)	0.5183 (0.4389,0.6120)
*α*_45_	0.2242 (0.1686,0.2980)	0.2523 (0.1936,0.3287)
*α*_46_	0.0361 (0.0171,0.0763)	0.0033 (0.0149,0.0742)
*α*_47_	0.0382 (0.0225,0.0647)	0.0541 (0.0352,0.0833)
*α*_54_	0.5482 (0.4651,0.6463)	0.5164 (0.4356,0.6123)
*α*_56_	0.0904 (0.0622,0.1316)	0.0906 (0.0625,0.1313)
*α*_57_	0.0357 (0.0211,0.0605)	0.0288 (0.0158,0.0524)
-2xLL	3969.72	3941.971

The results from Table 2 show narrow confidence intervals which is an indication that the suggested continuous time Markov model gives a precise estimate of the data. Results from Table 2 show that transitions to better CD4 cell count states are higher than transitions to worse CD4 cell count states which is an indication of efficacy of ART. The model with outliers has got a higher log-likelihood than the model without outliers as expected since the model with outliers has got a greater dimension. A further analysis on the transition intensities was also done for each of the CD4 baseline (CD4BL) and WHO stage baseline (WHOSBL) levels coded as follows:

$$ \mathrm{CD}4\mathrm{BL}=\left\{\begin{array}{llllll}\mathbf{1};& & & CD4& >& 750\\ {}\mathbf{2};& 500& <& CD4& \le & 750\\ {}\mathbf{3};& 350& <& CD4& \le & 500\\ {}\mathbf{4};& 200& <& CD4& \le & 350\\ {}5;& & & CD4& \le & 200\end{array}\kern1em \mathrm{and},\kern0.6em \mathrm{WHOSBL}=\left\{\begin{array}{ll}1;& Asymptomatic\\ {}2;& Mild symptoms\\ {}3;& Advanced symptoms\\ {}4;& Severe symptoms\end{array}\right.\right. $$

The results are shown in Appendix 2 and 3 for CD4BL and WHOSBL respectively. The results from Appendix 2 show that transition rates to CD4 recovery (2 to 1, 3 to 2, 4 to 3 and 5 to 4) were high for patients who initiated therapy when their CD4 baseline level was well above 350 per mm^3^. These rates of CD4 recovery decrease with as the CD4 cell count at treatment initiation decrease with a baseline CD4 cell count below 200 per mm^3^ recording the lowest rates of CD4 recovery. The results from Appendix 3 show that regardless of the WHO stage baseline, transition rates to CD4 recovery are higher than transition rates to CD4 deterioration. The rates of CD4 recovery are the highest for transitions from state 5 to state 4. Transition rates to state 6 (death) are the highest for those individuals who had severe HIV symptoms (WHOBL = 4) and these intensities decrease as the symptoms decrease from severe to asymptomatic levels.

### Expected holding times

The expected holding time in each state also known as the mean sojourn time describes the average time an individual spends in each state in a single stay before he/she makes a transition to another state. The mean sojourn time in each state *i* for *i* = 1, 2, …, 5, is estimated as $\frac{1}{\lambda_i}$, where ${\lambda}_i={\sum}_{i\ne j}{\alpha}_{ij}$ is the total force of transition out of state *i*. For example, the expected holding time in state 1 is 1/(0.887 + 0.1016 + 0.1418) ≈ 0.8844 as shown in Table 3 below:

**Table 3:** Expected holding times in each state

*i*	Estimates	SE	L	U
1	0.8844741	0.12602569	0.6689603	1.169418
2	0.8826571	0.09158707	0.7202261	1.081721
3	1.2482706	0.09636263	1.0729973	1.452175
4	1.2077295	0.07925375	1.0201303	1.331720
5	1.5728163	0.11539878	1.3621492	1.816065

Results from Table 3 show estimates of the holding time, the standard error (SE), the lower bound (L) and the upper bound (U) for each of the transient state *i*. From the results, if an individual is in state 5 (corresponding to a CD4 count below 200cell/mm^3^) he spends more time in that state before making a transition to other states. This could be due the time taken by an individual to respond to treatment since state 5 is the worst state in HIV/AIDS progression.

### The jump chain

This is when a Markov process is observed at the times it makes transitions to a new state. In other words a jump chain is a stochastic matrix *R* of probabilities where each row sums to one, on the state space *X*_*t*_, which gives the conditional probability of the next state an individual goes to after leaving state *i*. If *α*_*ii*_ > 0 then given that there is a jump to a different state, it means we never stay in state *i*, we make a jump out resulting in having *R*_*ii*_ = 0 and if *α*_*ii*_ = 0 then we never leave state *i* meaning that *R*_*ii*_ = 1 (States 6 and 7). The computed matrix of probabilities of each state being next (also known as the jump chain), together with the mean sojourn times in each state, fully define a continuous-time Markov model. This is a more intuitively meaningful description of a model than the transition intensity matrix. The matrix for the probabilities that the next state after state *i* is state *j* is approximated as ${p}_{ij}=\frac{\alpha_{ij}}{\lambda_i}$ , for each *i* and *j* such that *i* ≠ *j*. *α*_*ij*_ is the force of transition from state *i* to state *j* and *α*_*ii*_ is the total force of transition out of state *i*. For example, ${p}_{12}=\frac{\alpha_{12}}{\lambda_1}=\frac{0.8872}{0.8872+0.1016+0.1418}=0.7847$, as shown in the matrix below. The results are shown Table 4 below:

**Table 4:** Probability of each State being next (***R***_***ij***_)

	To	1	2	3	4	5	6	7
From	1	0	0.7847	0	0	0	0.0899	0.1254
	2	0.4575	0	0.4377	0	0	0.0715	0.0333
	3	0	0.5282	0	0.4149	0	0.0181	0.0388
	4	0	0	0.6041	0	0.294	0.0388	0.0631
	5	0	0	0	0.8123	0	0.1425	0.0451
	6	0	0	0	0	0	1	0
	7	0	0	0	0	0	0	1

The results from Table 4 show that *R*_*i*, 1 − 1_ > *R*_*i*, *i* + 1_, which shows that the probability of jumping to a better state is higher than the probability of jumping to a worse state. This is more pronounced for individuals in state 5 where the probability of jumping to state 4 (recovery) is 0.8123 which is very high compared to probability of making a jump to state 6. This is an indication of the effectiveness of treatment. Probability of the death state being next is the highest for those patients with CD4 counts less than 500. These probabilities increase with the decreasing number of CD4 counts.

### Forecast of the total length of stay in each state

We need to forecast the total time spent in the good states and the bad states by individuals who are on HIV treatment before death or withdrawal from the study. Estimates of the forecasted total lengths of time spent in each state *j* between two future time points *t*_1_ and *t*_2_ are estimated using the formula:

$$ {L}_j=\underset{t_1}{\overset{t_2}{\int }}{P}_{ij}(t) dt $$

where *i* is the state at the start of the process, which defaults to 1. The results are shown below:

**Table:** 

State1	State2	State3	State4	State5	State6	State7
8.988960	8.806075	7.767124	3.520485	1.153648	Inf	Inf

The results show that each individual is forecasted to spend approximately 8.99 half years in state 1, 8.8 half years in state 2, 7.77 half years in state 3, 3.52 half years in state 4 and finally 1.153 half years in state 5. These results show that HIV positive individuals on treatment are expected to spend more time in good states compared to the time spent in bad states.

Percentage prevalence for the model without covariates.

**Fig. 3:**
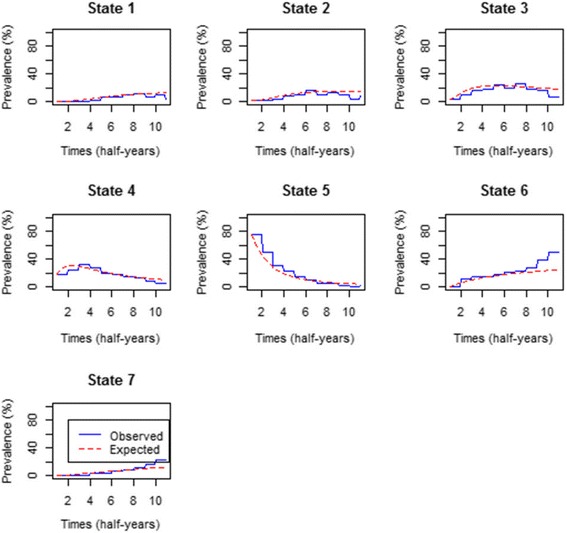
Comparison of observed and expected prevalence from the time-homogeneous model without covariates

Using the fitted time-homogeneous Markov model, the percentage prevalence were plotted to compare the expected values with the observed values. The results are shown in Fig. 3 below:

The results from Fig. 3 show that for the state *i* = 1, …, 6 the expected prevalence fit the observed data perfectly well except for the withdrawal state where the expected prevalence overestimate the observed. The plots further show a sharp decrease on state 5 percentage prevalence with the fitted model, underestimating the model for observed data up to time = 7 half years. The percentage prevalence for the death state is increasing at a slow rate and from time = 2 half years to time = 8 half years the percentage prevalence is stable.

### Effects of covariates on transition intensities

A continuous-time Markov model for the effects of covariates; Age, CD4BL, VLBL, WSBL, Reaction, DTB, TBB4 and Gender is fitted. Identification of covariates that have a significant contributory effect is done by entering each covariate one after the other and performing the likelihood ratio test in comparison to the model without covariates. All the other variables proved to be significant to the progression except for the variable gender which could not be eliminated because of its demographic importance. The baseline transition intensities $\left({\alpha}_{ij}^{(0)}\right)$ relate to the transitions from state *i* to state *j*. Baseline transition intensities and linear effect of each of the covariates is estimated and the results are shown in two separate Table 5 and Appendix 1 respectively:

**Table 5:** Baseline intensities and their corresponding confidence intervals for the covariate effects

(*i*, *j*)	Intensities (*α*_*ij*_)	B.L. Intensities $\left({\alpha}_{ij}^{(0)}\right)$
(1, 2)	0.8872 (0.6355,1.2390)	0.5030 (0.4277,0.8612)
(1, 6)	0.1016 (0.0541,0.1907)	0.0200 (0.0100,0.4641)
(1, 7)	0.1418 (0.0843,0.2386)	0.0175 (0.009,0.4934)
(2, 1)	0.5183 (0.3723,0.7217)	0.3900 (0.3846,0.6863)
(2, 3)	0.4959 (0.3729,0.6593)	0.4440 (0.2973,0.5550)
(2, 6)	0.0811 (0.0468,0.1404)	0.0111 (0.0073,0.2018)
(2, 7)	0.0377 (0.0167,0.0850)	0.0116 (0.001,0.0189)
(3, 2)	0.4231 (0.3451,0.5186)	0.3760 (0.3252,0.5010)
(3, 4)	0.3324 (0.2605,0.4241)	0.2333 (0.2076,0.3641)
(3, 6)	0.0145 (0.0047,0.0444)	0.0063 (0.00367,0.1287)
(3, 7)	0.0311 (0.0166,0.0584)	0.0095 (0.00285,0.3149)
(4, 3)	0.5183 (0.4389,0.6120)	0.5600 (0.4485,0.6284)
(4, 5)	0.2523 (0.1936,0.3287)	0.2300 (0.1522,0.2785)
(4, 6)	0.0033 (0.0149,0.0742)	0.0070 (0.0049,0.4210)
(4, 7)	0.0541 (0.0352,0.0833)	0.0084 (0.00589,0.1547)
(5, 4)	0.5164 (0.4356,0.6123)	0.5020 (0.4450,0.6297)
(5, 6)	0.0906 (0.0625,0.1313)	0.0198(0.00662,0.1456)
(5, 7)	0.0288 (0.0158,0.0524)	0.0055 (0.001280,3.936)
-2xLL	3941.971	3699.259

The fitted time homogeneous model with covariates has -2xLL = 3699.259, which represents an improvement of 242.712 compared to the model without covariates. A Likelihood ratio test is performed to compare the two nested models that were fitted, the one without covariates and the other with covariates. The value of the $LRT=-2 lo{g}_e\left(\frac{L_s\left(\widehat{\theta}\right)}{L_g\left(\widehat{\theta}\right)}\right)$ where ${L}_s\left(\widehat{\theta}\right)$ is the simple model (no covariates) and ${L}_g\left(\widehat{\theta}\right)$ is the general model (with covariates). A likelihood ratio test statistic of 1770.618 is compared to a *χ*^2^ distribution with 144 degrees of freedom. The test was performed and the results are shown below:

**Table:** 

	−2*logLR*	*Df*	*p-value*
*with.covariates*	1770.186	144	10^−4^

The results show that the model with covariates fits significantly better than the model without covariates.

#### Hazard ratios of covariates on transition intensities

In this section the hazard ratios for each of the covariates; VLBL-viral load baseline, DTB-develop TB during treatment period, TBB4-develop TB before treatment, Gender, React-reaction to treatment, CD4BL-CD4 baseline, WSBL-WHO stage baseline and Age are estimated. The relationship between these covariates and the transition intensities is defined by the following equation:

$$ {\alpha}_{ij}\left(\boldsymbol{Z}\right)={\alpha}_{ij}^{(0)}\exp \left({\beta}_{ij}^{\hbox{'}}\boldsymbol{Z}\right),\kern0.75em i\ne j, $$

where ***Z*** = [VLBL, DTB, TBB4, Gender, React, CD4BL, WSBL, Age ] is a *k* = 8-dimensional vector of covariates and *β*_*ij*_ is a vector of *k* regression parameters relating the instantaneous rate of transitions from state *i* to state *j* to the covariates ***Z*** and baseline intensities ${\alpha}_{ij}^{(0)}$ relating to the transition from state *i* to state *j* as shown in Table 5 above. Estimates of *β*_*ij*_’s, regression coefficients, were calculated and the results are shown in Appendix 1. The regression coefficients can be interpreted similarly to those in the proportional hazards regression model [19]. The results are shown in Table 6.

**Table 6:** Hazard ratios for the covariates on intensities

(*i*, *j*)	VLBL	DTB	TBB4	Gender	React	CD4BL	WSBL	Age
(1, 2)	0.69	2.29	0.31	2.04	4.72	1.17	0.55	1.45
(1, 6)	1.57	0.87	1.11	1.55	0.60	1.30	1.11	0.83
(1, 7)	1.01	0.96	1.00	1.26	0.68	0.92	0.56	0.40
(2, 1)	0.48	1.14	0.76	1.33	1.46	1.17	0.74	2.63
(2, 3)	0.92	1.98	0.67	6.46	0.67	1.37	0.26	0.40
(2, 6)	1.12	0.96	1.69	1.45	0.54	0.997	1.16	1.10
(2, 7)	1.30	1.19	1.92	0.93	0.73	1.33	0.79	0.70
(3, 2)	0.68	1.58	1.10	2.35	2.08	0.69	0.75	0.83
(3, 4)	0.42	2.55	0.53	1.04	0.55	1.57	1.06	0.89
(3, 6)	0.92	1.17	2.03	1.20	0.59	1.05	1.32	1.77
(3, 7)	1.34	0.86	2.34	0.72	0.20	1.20	0.54	2.06
(4, 3)	1.52	1.72	0.86	0.61	1.02	0.27	0.84	1.08
(4, 5)	0.74	2.25	1.65	1.36	0.65	1.05	0.46	0.89
(4, 6)	1.31	1.09	1.09	1.59	0.22	1.01	1.40	1.96
(4, 7)	1.56	1.17	1.88	0.58	0.36	1.02	0.52	2.18
(5, 4)	0.51	1.86	1.02	0.81	1.32	0.61	0.40	0.63
(5, 6)	0.92	0.65	0.41	2.10	0.06	1.003	2.09	2.60
(5, 7)	0.97	1.20	0.88	1.19	0.66	0.87	0.40	2.31

The -2xLL for the model fitted in Table 6 is 3699.259. The results show that the strongest predictor of transition from state 1 to 2 is a negative reaction to treatment, which has a hazard ratio of 4.715. This means that patients who developed some form of reaction were over 4 times more likely to transit from a level of CD4 ≥ 750 to a level of 500 ≤ CD4 < 750 than patients who did not react to treatment. However, from all the other states, hazard ratios for the patients who reacted to treatment are higher for immune recovery than for immune deterioration.

The strongest predictor of immune deterioration from a CD4 level between 350 and 500 to a CD4 level between 200 and 350 (3 to 4) is developing TB during treatment, with a hazard ratio of over 2. Developing TB is also the strongest predictor of immune deterioration from 4 to 5, with a hazard ratio also greater than 2. This means that TB is the major cause of further immune deterioration when the immune system is too weak. Hence the recommendation that HIV patients should continuously have their TB status checked. Those individuals who had TB before enrolment had the strongest predictor for the transition from state 3 to state 6. These patients had a hazard ratio of over 2 times more likely to die from state 3 than those who were enrolled without having TB. However, for these individuals, transitions to better states were generally higher than transitions to worse states for almost all states.

A hazard ratio of 6.46 for the predictor variable male shows that males were over 6 times more likely to transit from state 2 to 3 than their female counterparts. The hazard ratios of males from a bad state to a better state are less than 1, which is an indication that males are less likely to respond to treatment compared to females.

The hazard ratios for the transitions to a better state for patients who were enrolled with CD4 counts below 350 are less than one, but hazards to worse states are greater than one, an indication that starting treatment when the CD4 levels are below 350 retards immune recovery. The transitions to the death state for individuals who started treatment when they were on the WHO stage of 4 are all more than one, meaning that starting treatment with a WHO stage of 4 is a leading cause of being absorbed in the death state.

#### Percentage prevalence for the model with covariates

**Fig. 4:**
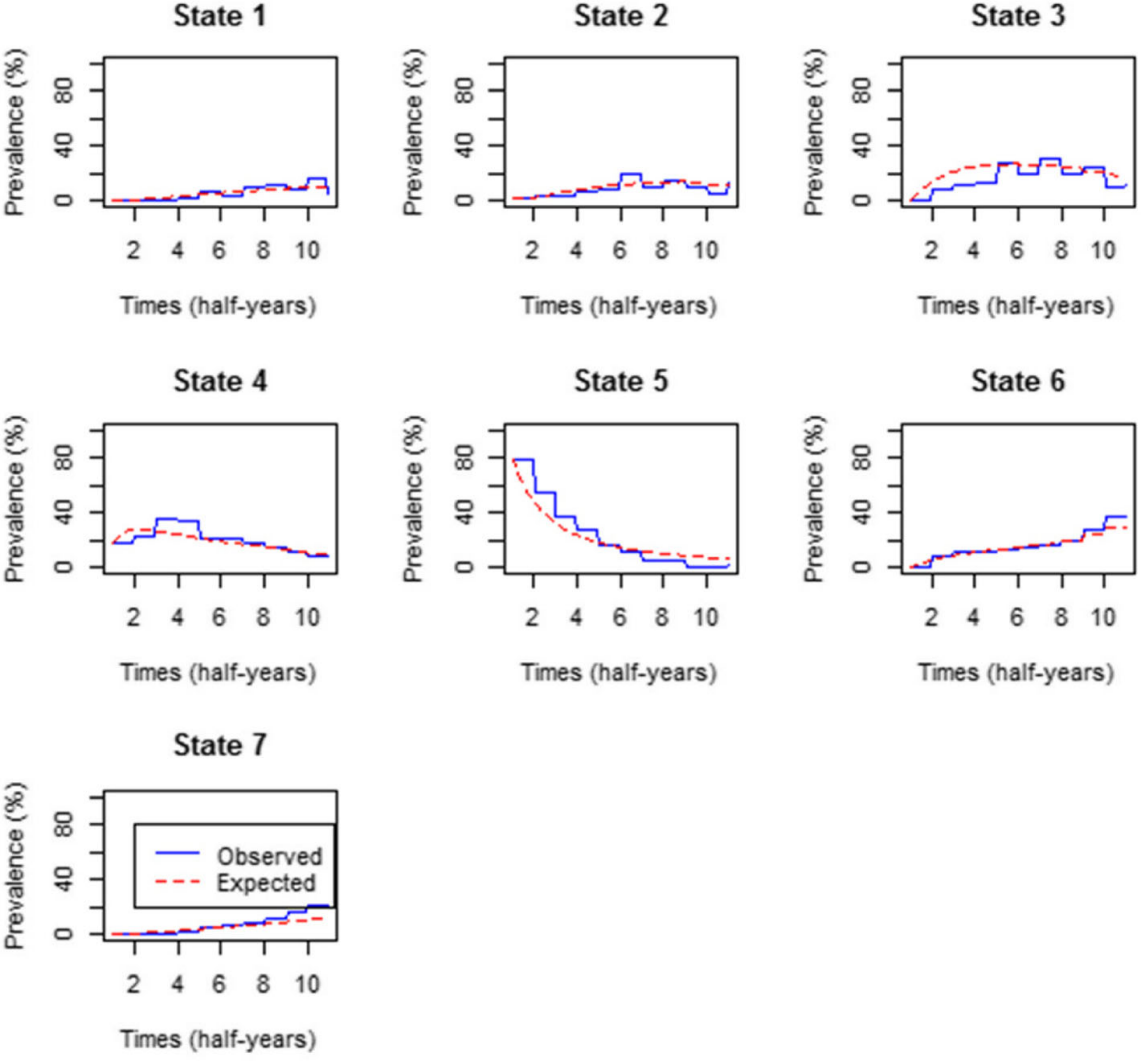
Comparison of observed and expected prevalence from the time-homogeneous model with covariates

The prevalence for the model with covariates were plotted to examine areas of poor fit of the time-homogeneous model with covariates. The plots are shown in Fig. 4.

Figure 4 confirms that the inclusion of covariates on the model improves the fitness of the model since the expected prevalence is now perfectly closer to the observed prevalence for all states than the model without covariates.

## Conclusion

In this paper a continuous-time homogeneous Markov model is fitted to explore predictors of HIV/AIDS progression for patients on antiretroviral therapy. A continuous-time homogeneous model is fitted with and without covariates and comparison of these two models is done using the likelihood ratio test. Parameters that define progression of HIV/AIDS were estimated and these include transition intensities, mean sojourn times and probability of each state being next or jump chains. The fitted model is used to analyse the effects of the covariates on the transition intensities. These covariates were reaction to treatment, development of TB during treatment and gender among others.

Results from the likelihood ratio test show that the model with covariates provides a better fit than the model with no covariates with a *p*-value =10^{−4}^. The results show that transition rates to immune recovery are generally higher than the transition rates to immune deterioration. However, the results show that the strongest predictor of immune deterioration from state 1 (CD4 cell count greater than 750) to state 2 (CD4 cell count between 500 and 750) is reaction to treatment. These patients are 4 times more likely to transit from state 1 to state 2 than those who did not react to treatment.

Patients who developed TB during the course of treatment have higher chances of immune deterioration than immune recovery compared to those who did not develop any TB co-infection. These incidences are quite high for transition from state 4 (CD4 cell count between 200 and 350) to state 5 (CD4 cell count below 200). For these states the immune system is still weak. As a result patients on antiretroviral drugs should consistently be screened for TB co-infection. Patients who had initially been diagnosed with TB before commencement of ART recover better from HIV/AIDS disease except that transitions to death for patients with CD4 cell count between 350 and 500 cells/mm^3^ are two times higher than that of patients who were not initially diagnosed with TB.

From this cohort, transitions to bad states are higher for males than for their female counterparts. This is quite pronounced on transitions from state 2 (CD4 cell count between 500 and 750) to state 3 (CD4 cell count between 350 and 500) where the hazards for males are 6 times that of females. This result is consistent with the findings from Maskew and others, they discovered that men gain fewer CD4 cell counts than did women [20]. An assessment of published studies by Castillo and others [21] from both resource-limited and resource-rich countries suggest an improved survival outcomes for females than males. However, the studies they assessed do not show a clear sex disparity in the disease progression or in treatment effects of viral suppression and immunologic recovery.

The results from the fitted model show that the rates of immune recovery were much higher than the rates of immune deterioration which is an indication of effectiveness of treatment. Patients who started treatment when their CD4 baseline was at least 350 had higher rates of immune recovery than those who had a lower CD4 baseline. This result is commensurate with the findings from Moore and Keruly who also discovered that patients with baseline CD4 cell count above 350 cells/mm^3^ returned to nearly normal CD4 cell count after 6 years [22]. The probability of dying increases with decreasing CD4 count of the individual at enrolment. This is supported by the findings of [23–25], who also concluded that being in the AIDS defining stage leads to the highest probability of reaching the death state.

The mean sojourn times revealed that patients take longer time in the AIDS defining states (CD4 cell count below 200) before they move to the other states. Research has also shown that CD4 cell count rises gradually despite the suppressed viral load particularly in older patients. Hence, there is need to use both CD4 cell count and viral load in monitoring the efficacy of treatment. The younger people below the age of 40 have higher chances of immune recovery than the older ones. This finding is supported by some previous studies who concluded lower mean CD4 increases for older patients than younger patients [20, 26]. Alioum and others further argued that this could be caused by the fact that older subjects may have a reduced capacity to generate CD4 cells in response to the viral killing [10].

Although continuous time Markov models can handle multiple or recurrent outcomes compared to the Kaplan Meier analysis and Cox proportional hazards models, the assumption of constant hazard function that is frequently unrealistic [27] and puts limitations on the disease history behaviour [28], especially on HIV/AIDS progression for patients on ART. Some studies have shown that if a patient responds well to treatment and manages to achieve viral load suppression within the first 6 months, that patient is likely to continue responding well to treatment [29]. This goes against the Markov and memoryless properties of the models. Thus a limitation in the application of time homogeneous Markov processes.

## Appendix 1

**Table 7:** Linear effects of covariates on transition intensities

Param.	VLBL	CD4BL	WSBL	React	DTB	TBB4	Gender	Age
*β*_12_	−0.37	0.15	−0.59	1.55	0.83	−1.17	0.7118	0.37
*β*_16_	0.45	0.27	0.101	−0.52	−0.14	0.11	0.4379	−0.18
*β*_17_	0.0071	−0.088	−0.58	−0.39	−0.039	0.00086	0.23	−0.90
*β*_21_	−0.73	0.156	−0.31	0.38	0.13	−0.28	0.29	0.97
*β*_23_	−0.84	0.31	−1.35	−0.40	0.68	−0.39	1.87	−0.92
*β*_26_	0.112	−0.0028	0.150	−0.63	−0.044	0.53	0.37	0.100
*β*_27_	0.26	0.29	−0.24	−0.32	0.17	0.65	−0.077	−0.36
*β*_32_	−0.39	−0.37	−0.28	0.73	0.46	0.096	0.86	−0.18
*β*_34_	−0.86	0.45	0.057	−0.59	0.94	−0.63	0.036	−0.116
*β*_36_	−0.085	0.047	0.28	−0.53	0.16	0.71	0.18	0.57
*β*_37_	0.29	0.181	−0.24	−1.63	−0.15	0.85	−0.33	0.72
*β*_43_	0.42	−1.32	−0.17	0.020	0.54	−0.15	−0.49	0.080
*β*_45_	−0.305	0.049	−0.77	−0.43	0.81	0.50	0.31	−0.12
*β*_46_	0.27	0.0079	0.34	−1.50	0.087	0.089	0.46	0.67
*β*_47_	0.45	0.020	−0.66	−1.01	0.16	0.63	−0.55	0.78
*β*_54_	−0.66	−0.501	−0.91	0.27	0.62	0.022	−0.22	−0.46
*β*_56_	−0.079	0.0030	0.74	−2.83	−0.43	−0.89	0.74	0.95
*β*_57_	−0.028	−0.141	−0.92	−0.41	0.19	−0.13	0.18	0.84

## Appendix 2

**Table 8:** Transition intensities for each CD4 baseline

Transition	2	3	4	5
1 to 2	3.1280	6.3180	12.760	25.780
1 to 6	0.0125	0.0203	0.0330	0.0537
1 to 7	0.0389	0.0560	0.0807	0.1163
2 to 1	0.6644	0.5991	0.5407	0.4870
2 to 3	0.2842	0.4054	0.5783	0.8249
2 to 6	0.0176	0.0184	0.0194	0.0203
2 to 7	0.0227	0.0278	0.0341	0.0417
3 to 2	0.2450	0.1658	0.1123	0.0760
3 to 4	0.2749	0.1781	0.1153	0.0747
3 to 6	0.0079	0.0088	0.0098	0.0108
3 to 7	0.0347	0.0332	0.0318	0.0305
4 to 3	1.3990	0.8116	0.4708	0.2731
4 to 5	0.8935	0.6913	0.5348	0.4138
4 to 6	0.0110	0.0011	0.0103	0.0099
4 to 7	0.0496	0.0510	0.0524	0.0539
5 to 4	13.140	5.3300	2.1620	0.8774
5 to 6	0.0192	0.0167	0.0145	0.0126
5 to 7	0.3747	0.2259	0.1362	0.0821

## Appendix 3

**Table 9:** Transition for the WHO Stage Baseline Line

Transition	1	2	3	4
1 to 2	0.3143	0.1289	0.0529	0.0217
1 to 6	0.0044	0.0041	0.0038	0.0035
1 to 7	0.0139	0.0103	0.0076	0.0057
2 to 1	0.8460	0.8758	0.9066	0.9384
2 to 3	0.1250	0.1119	0.1001	0.0896
2 to 6	0.0171	0.0182	0.0194	0.0207
2 to 7	0.0139	0.1286	0.0119	0.0109
3 to 2	0.8116	1.2320	1.8700	2.8390
3 to 4	0.8856	1.1970	1.6170	2.1850
3 to 6	0.0072	0.0081	0.0091	0.0102
3 to 7	0.0305	0.0245	0.0197	0.0159
4 to 3	4.5930	5.0750	5.6070	6.1950
4 to 5	1.2360	1.0240	0.8484	0.7028
4 to 6	0.0139	0.0165	0.0195	0.0232
4 to 7	0.0325	0.0226	0.0157	0.0109
5 to 4	67.220	56.630	47.710	40.190
5 to 6	0.0390	0.0596	0.0912	0.1394
5 to 7	0.5792	0.3253	0.1827	0.1026
